# Interstep Variations of Stairways and Associations of High-Contrast Striping and Fall-Related Events: Observational Study

**DOI:** 10.2196/60622

**Published:** 2025-01-08

**Authors:** Sara A Harper, Chayston Brown, Shandon L Poulsen, Tyson S Barrett, Christopher J Dakin

**Affiliations:** 1 Department of Kinesiology and Health Science Sorenson Legacy Foundation Center for Clinical Excellence Utah State University Logan, UT United States; 2 Kinesiology Department University of Alabama in Huntsville Huntsville, AL United States; 3 Highmark Health Research Institute Pittsburgh, PA United States

**Keywords:** stairs, stairway safety, riser height, tread depth, horizontal-vertical illusion, fall risk, fall prevention, videos, Monte Carlo simulation, public health, vision-based strategy, health promotion, adults, geriatric

## Abstract

**Background:**

Interstep variations in step riser height and tread depth within a stairway could negatively impact safe stair negotiation by decreasing step riser height predictability and, consequently, increasing stair users’ fall risk. Unfortunately, interstep variations in riser height and depth are common, particularly in older stairways, but its impact may be lessened by highlighting steps’ edges using a high-contrast stripe on the top front edge of each step.

**Objective:**

This study aimed to determine (1) if fall-related events are associated with greater interstep riser height and depth variations and (2) if such fall-related events are reduced in the presence of contrast-enhanced step edges compared with a control stairway.

**Methods:**

Stair users were video recorded on 2 public stairways in a university building. One stairway had black vinyl stripes applied to the step’s edges and black-and-white vertical stripes on the last and top steps’ faces. The stairway with striping was counterbalanced, with the striped stairway than a control, and the control with stripes. Each stair user recorded was coded for whether they experienced a fall-related event. A total of 10,000 samples (observations) of 20 fall-related events were drawn with 0.25 probability from each condition to determine the probability of observing a distribution with the constraints outlined by the hypotheses by a computerized Monte Carlo simulation.

**Results:**

In total, 11,137 individual stair user observations had 20 fall-related events. The flights that had 14 mm in interstep riser height variation and 38 mm in interstep depth variation were associated with 80% (16/20) of the fall-related events observed. Furthermore, 2 fall-related events were observed for low interstep variation with no striping, and 2 fall-related events were observed during low interstep variation with striping. A total of 20 fall-related events were observed, with 4 occurring on flights of stairs with low interstep variation. For stairs with high variability in step dimensions, 13 of 16 (81%) fall-related events occurred on the control stairway (no striping) compared with 3 of 16 (19%) on the high-contrast striping stairway. The distribution of fall-related events we observed between conditions likely did not occur by chance, with a probability of 0.04.

**Conclusions:**

These data support the premise that a vision-based strategy (ie, striping) may counteract fall risk associated with interstep riser height and tread depth variation. Possibly, perception and action elicited through the horizontal-vertical illusion (striping) may have a positive impact on the incidence of fall-related events in the presence of high interstep riser height and depth variation. The findings of this study suggest that contrast enhancement (ie, striping) may be a simple and effective way to reduce the risk of falls associated with interstep variation, highlighting the potential for this approach to make a significant impact on fall prevention efforts.

## Introduction

When approaching a stairway, stair users seemingly anticipate uniformity in the step riser height and tread depth [1,2]. However, this assumption may lead to fewer attentional resources being allocated to estimating these metrics, potentially compromising the safe negotiation of the stairway. Unfortunately, interstep riser height and tread depth variations are common. Often described as dimensional uniformity in building code, stair riser heights and tread depths shall be of uniform size and shape. The tolerance between the largest and smallest riser height, or tread depth, shall not exceed 3/8 inch in any flight of stairs. In our investigation, the range of dimension uniformity is referred to as interstep variation. Interstep variations, as small as 6 mm, between adjacent stair risers or treads can disrupt step cadence and increase the risk of accidents or falls [3]. One strategy to mitigate this risk involves applying a horizontal-vertical illusion and black stripes to the top front edge of each step, which could potentially decrease the frequency of slips, trips, and falls [4-6]. Recent research has demonstrated that adding a high-contrast stripe along the top front edge of each step [4,7-11] can lead to increases in heel clearance above the step. Similarly, adding vertical monochrome striping to the faces of the bottom and top steps can also enhance vertical foot clearance [4,5,12,13].

Although the exact mechanism behind these interventions is not fully understood, it is possible that they increase step height by drawing more attention to each step’s edge [7] or by creating a horizontal-vertical illusion, which makes the steps appear taller than they actually are [14,15]. Ultimately, either mechanism may decrease the likelihood of a slip, trip, or fall on stairs with high interstep variation. We, therefore, hypothesized that when comparing 2 flights of stairs with similar interstep variability, the stairs with vertical monochrome striping and tread edge highlighters would record fewer fall-related events than stairs without this intervention. In addition, stairs with greater interstep variation in riser heights and tread depths would generally record more fall-related events than stairs with less interstep riser height and tread depth variability, but this effect would be lessened with the addition of monochrome striping and tread edge highlighters.

To test our hypotheses, we estimated the probability of observing a range of fall-related event distributions that could plausibly occur by chance, given our hypotheses [high or low interstep riser height and tread depth variations and control or striping intervention conditions]. We codified our hypotheses in a Monte Carlo simulation using 4 constraints based on our hypotheses. First, there would be more fall-related events on control flights of stairs (without the contrast intervention) with greater interstep variation than those with lower interstep variation. Second, both control and intervention (with the contrast enhancement) flights of stairs with low interstep variation should observe a comparable frequency of fall-related events (ie, a difference of less than 2 fall-related events between conditions). Third, fewer (less than or equal to half the number of) fall-related events should be observed with high interstep variation intervention stairs compared with the high interstep variation control stairs. Finally, the relative difference in fall-related events between high and low interstep variation stair flights in the control condition should be greater than on the intervention flights.

## Methods

### Participants

This cross-sectional study took place on 2 public stairways on the Utah State University campus. Video capture occurred on public stairways, and most stair users appeared to be young adults.

As previously discussed, 11,137 individual stair user observations were coded and balanced across the stairway conditions (control and intervention) and stairways (East and West) [16]. There were 7458 (66.97%) feminine observations and 3679 (33.03%) masculine observations, where most observations (n=10,970, 98.5%) were in the age group of 18-40 years. Additional participant details were described previously [6]. Given the observational nature of this study in a public space, no screening was performed in advance. Eligibility criteria were as follows: (1) inclusion—visually appearing 18 years of age and older, captured within local time (eg, 7 AM-5 PM) and (2) exclusion—use of assistive walking devices (eg, crutches and walking boots), individuals that did not transverse the stairs, or involved unusual stairway behavior, as described by Harper et al [6] were documented and removed during the data cleaning phase.

### Protocol

High-resolution security cameras (8 megapixels, 4K Ultra HD, 3840×2160 resolution at 7 frames per second, Lorex cameras [Lorex Technology Inc]) were placed in the stairways to record stair users’ behavior.

### Intervention

High-contrast black vinyl film (Gerber High-Performance Series 220 vinyl film [Gerber Technology]) stripes (5.5 cm wide, 0.063-0.09 mm thick) were applied flush to the top front edge of each stair [4,9,12]. A total of 19 black-and-white vertical vinyl stripes (12 cycles/1 meter) were placed on the very bottom and top steps’ faces [4,9,12].

**Figure 1:**
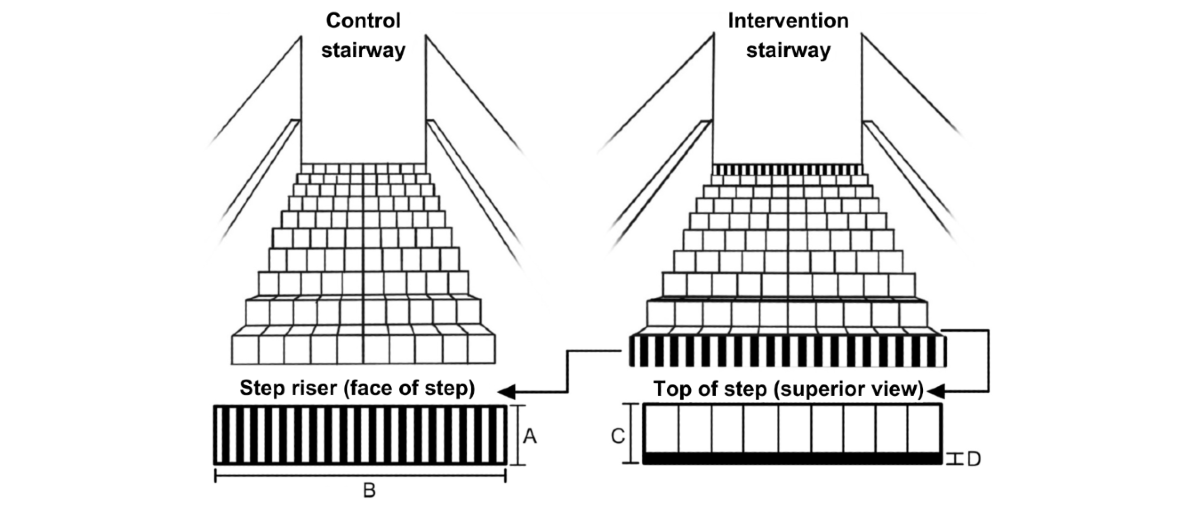
Control and interventional stairway conditions. Intervention conditions are further illustrated to show step riser (face of step) and top of step (superior view) vinyl stripes. Step riser height=step height. Stairway width=step tread depth. Vinyl striping depth. Adapted from Harper et al [6], which is published under Creative Commons Attribution 4.0 International License [17].

**Figure 2:**
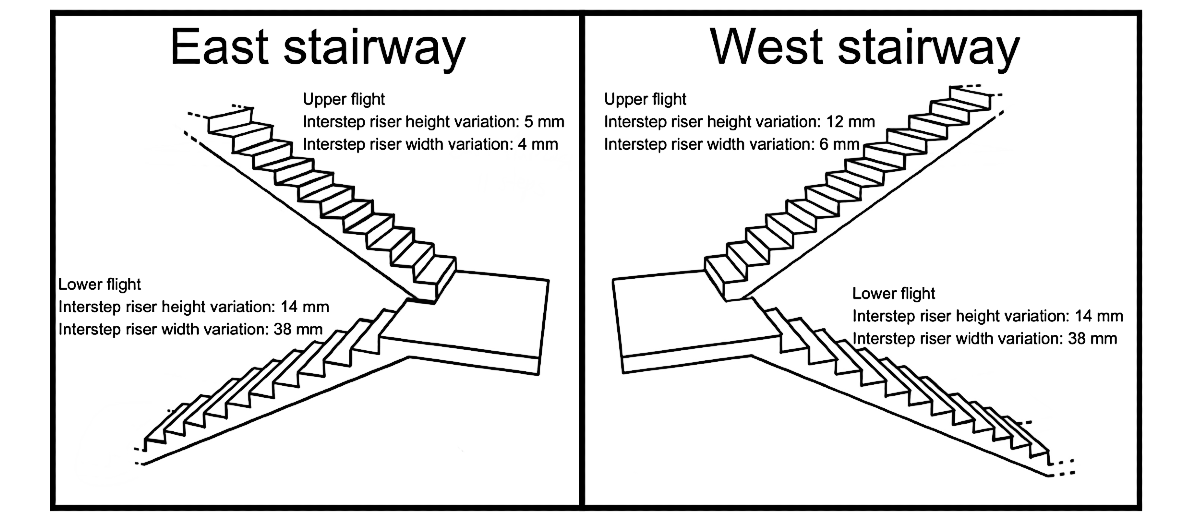
Real-world stairway design. East upper and lower stairway flights interstep riser height variation ranged between 5 mm and 14 mm, and interstep depth between 4 mm and 38 mm, respectively [6]. West upper and lower stairway flights’ interstep riser height variation ranged between 12 mm and 14 mm, as well as interstep tread depth variation of 6 mm and 38 mm respectively, are shown [6]. Adapted from Harper et al [6], which is published under Creative Commons Attribution 4.0 International License [17].

Figure 1 [6] depicts the combined striping intervention. Stairway interstep riser height and depth variations were measured across every step, from the middle part of the step (Figure 2) [6].

In total, 48 steps were observed across 4 flights of stairs. The control stairway was unaltered and used to compare with the intervention stairway. Halfway through data collection, the intervention (striped) and control stairways were counterbalanced.

### Measures

**Figure 3:**
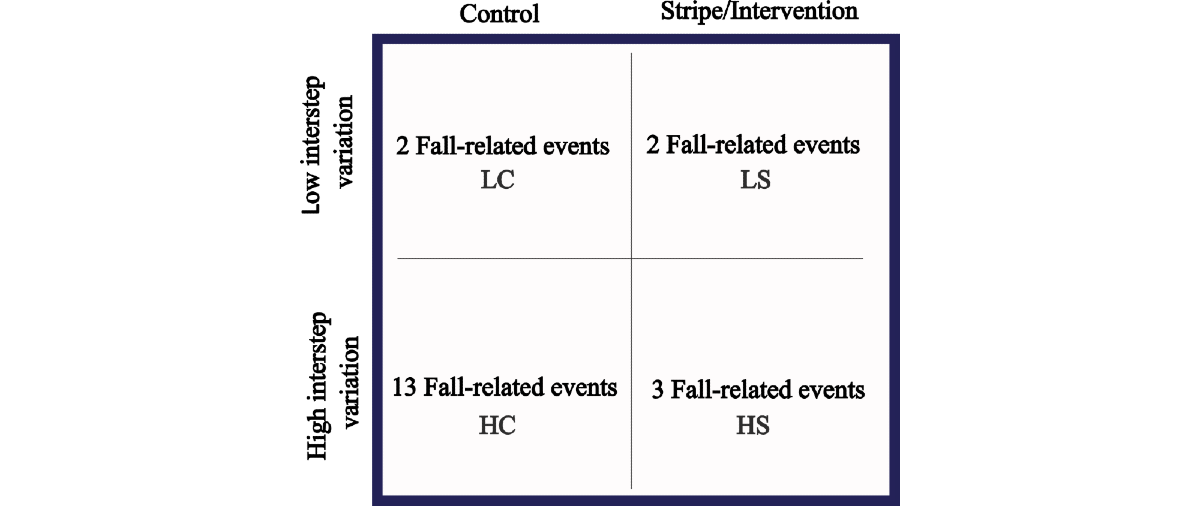
The probability of observing a range of distributions of fall-related events. The following assumptions evaluated the observed fall-related event distribution. HC: high interstep variations, control condition; HS: high interstep variations stripe intervention; LC: low interstep variations, control condition; LS: low interstep variations, stripe intervention.

Each outcome variable was assessed by stairway location (East and West) and condition (intervention and control), as well as by stairway flight (lower and upper; Figure 3).

In total, 4 assumptions were used to code our hypotheses based on our a priori knowledge of the total number of fall-related events recorded (n=20): (1) the number of fall-related events high interstep variations, control condition>low interstep variations, control condition; (2) the difference between low interstep variations, stripe intervention and low interstep variations, control condition ≤2 fall-related events; (3) the number of falls in high interstep variations, control condition will be ≥2 times of high interstep variations stripe intervention; and (4) the difference between low interstep variations, control condition and high interstep variations, control condition > the difference between low interstep variations, stripe intervention and high interstep variations stripe intervention. The probability of a distribution meeting these assumptions occurring by chance is approximately 0.04 with a sample size of 20.

### Data Sources

We coded stair users’ navigation direction (ascent and descent) and the presence of a fall-related event. As described previously, a fall-related event was coded if an observed stair user experienced a relatively subtle trip or slip, with minimal recovery action, through a complete loss in balance, resulting in a fall [6]. If a fall-related event occurred, the stairway and step number (starting from the bottom to the top) were recorded. To assess research bias or intercoder reliability, each week, one of the researchers would randomly select and evaluate 10% of the data collected for that week. If an error was present, a second coder reviewed all data recorded by the first coder for the week in question, and the second coder made a determination on the final coding record [6]. Data were encoded using Microsoft Excel.

### Statistical Analysis

Data are presented as mean (SD) as well as count (percentage) of observed results. The distribution of fall-related events was assessed across stairway flights (interstep variations) and conditions (control and intervention) using a 0.5-inch threshold (approximately 13 mm), given that 75% (80/101) of stair accidents occurred in stairways with interstep riser height variations of ≥0.5 inch [18]. A Monte Carlo simulation in Julia [19] was used to estimate the probability of observing the distribution of fall-related events defined by our hypotheses by chance. Specifically, 10,000 samples (observations) of 20 fall-related events (refer to the Results section) were drawn with 0.25 probability from each condition to determine the probability of observing a distribution with the constraints outlined by the hypotheses through a computerized Monte Carlo simulation [20,21].

### Ethical Considerations

Ethical approval was obtained from the institutional review board of Utah State University (10773). As an observational study, participants did not give written consent. Given the observational nature, participants were not compensated to participate. The video recordings were taken in a public setting, and only the approved research team had access to the identifiable data. Therefore, our video data are not available as supplementary material.

## Results

Individuals who had any visible health-related conditions (eg, crutches and walking boots) were documented and removed before analysis. In addition, those who visually appeared under the age of 18 years were documented and removed before analysis. All steps were measured at the center of the steps. The average of the East and West stairways combined was 168 (SD 4) mm, with the average riser height of the West stairway steps being 171 (SD 3) mm, and the East stairway being 166 (SD 3) mm, independently. The average step tread depth for the East and West stairways was 328 (SD 8) mm, the average depth of the West stairway steps was 327 (SD 8) mm and the Easy stairway was 329 (SD 8) mm.

Of 20 observed fall-related events, 80% (n=16) of events were observed on the flights where interstep variations were the greatest (riser height ranged 14 mm and tread depth ranged 38 mm). In comparison, 4 of 20 fall-related events were observed on flights with lower interstep variation. Between East and West stairways, 7 of 20 (35%) fall-related events occurred on steps that had step riser heights greater than 1 SD from their mean including 2 fall-related events that occurred on the first step while ascending (step riser height=170 mm; flight mean 166, SD 3 mm), 3 events on the last step while descending the East, lower stairway (height=155 mm; mean 166, SD 3 mm), 1 event on the last step while ascending the East, upper stairway (height=170 mm; mean 166, SD 3 mm), and 1 event on the second-to-last step while ascending the West, lower stairway (height=173 mm; mean 171, SD 4 mm).

A total of 20 fall-related events were recorded. On flights where interstep riser height variation ranged 14 mm and step depth variation ranged 38 mm, 13 of 16 (81%) fall-related events occurred on the control stairway condition (no striping), compared with 3 of 16 (19%) on the striped intervention stairway. Finally, 2 fall-related events were observed for low interstep variation with no striping, and 2 fall-related events were observed during low interstep variation with striping.

The estimated probability of observing data that fit the range of distributions constrained by our hypotheses and using a sample size of 20 fall-related events, was approximately 0.04 (Figure 3). This result suggests that (1) interstep variations may be contributing to falls and (2) adding a striping intervention to the stairs may reduce the impact of interstep variations on fall-related events.

## Discussion

### Principal Results

We sought to assess the impact of interstep riser height and tread depth variations on fall-related events (eg, slips, trips, or falls) occurring on stairways to determine if fall risk increases with greater interstep variations but is reduced by adding high-contrast striping. The lower flights of stairs, which had interstep riser height variations that ranged 14 mm and interstep depth variations that ranged 38 mm, accounted for 80% (16/20) of the observed fall-related events, supporting the notion that stairways with greater interstep variations may be associated with a greater risk of fall-related events. Furthermore, 35% (7/20) of fall-related events were observed on steps where interstep riser height variation was greater than 1 SD from the flight mean. Together, these results suggest that flights of stairs with greater interstep riser height variation exhibit more fall-related events than flights with lower interstep variation. When high-contrast striping was added to flights of stairs with high interstep variation, there were fewer fall-related events observed over a similar time period (3/20 fall-related events in the intervention vs 13/20 fall-related events in the control condition). This result suggests the addition of high-contrast striping to stair flights with high interstep variation may reduce the number of fall-related events resulting from interstep variation.

### Comparison With Previous Work

#### Interstep Variations Increase Fall Risk

Interstep variations on stairways can have a profound negative impact on fall risk. Even minor interstep variations, such as a 6 mm variance in riser height [3], can disrupt step cadence and increase the likelihood of a fall, as can interstep variation in tread depth. Furthermore, a review of 80 stairway falls from 1992 to 2007 found that 60% (48/80) of riser height and 34% (27/80) of interstep depth variation were greater than 3/8 inch in a study by Cohen et al [7], and greater (3/8 inch compared with 0.5 inch) interstep variation that can disrupt cadence [3]. Francksen et al [22] found that adults could adjust their stepping behavior for increases in depth, but they could not adjust for interstep riser height variation over 10 mm. Stair-related fall injuries are also more commonly associated with interstep riser height variation than interstep tread depth variation [7]. Alternatively, the greater association of falls with interstep riser height variation could be due to the increased frequency of observing interstep riser heights compared with tread depth variations (eg, > 3/8 inch is observed more often in riser heights [48/80, 60%] than tread depths [27/80, 34%]) [7]. Nevertheless, additional research is needed to explore the extent and impact of interstep variation on stair-related falls. In this study, we observed that 80% (16/20) of fall-related events on stairs occurred when the interstep riser height variation was 14 mm and depth was 38 mm. Since interstep riser height and depth variations were both present in our observational design, we are unable to distinguish which of these factors may have had a greater role.

#### The Intervention Was Associated With Reduced Fall-Related Events With Interstep Variation

Broadly, our findings indicate that greater interstep variations are associated with an increased frequency of fall-related events. Importantly, the frequency of these fall-related events decreased when the intervention was present. Our data suggest that adding high-contrast stripes may reduce fall-related events on stairs when large interstep variations are present. Of the 20 fall-related events observed, 16 were on the flights of stairs with larger interstep variation, but only 19% (3/16) occurred when the intervention was present, whereas 81% (13/16) occurred when the intervention was absent.

#### Mechanisms Contributing to the Reduction in Fall-Related Events

The intervention may induce a horizontal-vertical illusion by the intervention. This occurs when horizontal and vertical lines of similar length are presented together (like the letter “T”), which results in an illusory sense that the vertical line is longer than the horizontal one. The intervention used in this study included black vinyl stripes applied to each step’s top front edge and black-and-white vertical stripes on the face of the first and last steps. Together, the abutting edges of the combined striping that formed “T”-like configurations could have contributed to an increased perceived step riser height [14], resulting in greater step heights. Indeed, previous research suggests that under similar experimental conditions, perceived step height is increased and is associated with an increase in the height of the step taken [12,13,15]. In further support for this mechanism, the horizontal-vertical illusion effect is reduced when only edge highlighters are present [13], suggesting that the vertical lines contribute to the increased step height and, perhaps, that the horizontal-vertical illusion is a primary mechanism contributing to the reduction in fall-related events we observed with the intervention. It is also possible that other intervention formats, such as those that could induce the Müller-Lyer illusion [23], could reduce fall-related events. The Müller-Lyer illusion occurs when the perceived length of a line is influenced by the orientation of arrow-like segments attached to its ends. Lines of the same length appear shorter or longer depending on whether the arrowheads at the ends point inward or outward. By manipulating the direction of the fins at the end of the lines, perhaps step height could be increased or decreased depending on the particular interstep variation. Recent outdoor observational research suggests that greater vertical foot clearance occurs when a “fins out” configuration is applied to a 2-step stairway [24], which is the illusion’s expected effect. Future research could examine whether a “fins in” configuration reduces step height.

By accentuating the steps’ edges, the intervention may draw attention to them, thereby enhancing the accuracy with which stair users can estimate the dimensions of each step and then compensate for irregularities. Future research could investigate whether the presence of striping increases awareness of interstep variation, thereby evaluating this potential mechanism. In addition, the novelty of the intervention itself may have drawn attention to the stairs. Schomaker and Meeter [25] suggest that novel visual stimuli, such as a change in contrast, may increase attention to the stairs [26]. It is worth noting that the striping intervention was installed several days before data collection began. It is likely that some stair users had exposure to the striping before the start of video recording and this early exposure could have reduced the novelty of the striping effect before data collection, thus reducing the drawing effect. We also expect that this effect would have worn off over time as many of the observed stair users traverse the stairs frequently due to regular classes in the building.

#### What Should Be Done About Excessive Interstep Variations

Maybe the most important challenge associated with observing increased fall-related events with greater interstep variations is what to do about it. Is the risk of falls sufficient to warrant widespread evaluation and enforcement of building codes? Although we do not provide recommendations here, conducting assessments, enforcing regulations during construction, and evaluating older stairways (likely to exhibit the greatest interstep variation) may reduce fall-related events, especially when considering cost-effectiveness. For older stairways, as used in this study (built in 1971, and at the time of construction, the 1967 Uniform Building Code [Sec 3305 (d)] that was in place in the United States required the maximum interstep variation in riser height and tread depth to be no more than 3/16 inch), adding an intervention like painting stripes might be the most cost-effective way (an estimated US $288 for the high-contrast striping used here) to reduce the impact of interstep variations (assuming the application or materials used to apply the striping do not themselves increase fall risk through reduced or increased friction, or materials peeling). While increased interstep variation is associated with a greater risk of fall-related events, there are cost-effective interventions that can help reduce this risk. By enforcing building codes and evaluating stairways for interstep variation, we can work toward creating safer environments for everyone.

### Limitations

While these results are promising, we acknowledge several limitations of this study. Even with over 10,000 observations, we only observed 20 fall-related events. A larger sample of falls would provide a more precise estimate of the differences between flights of stairs and strengthen the inferences that could be drawn from these data. Since most of the observations in this study were younger adults, future work should consider targeting older or clinical populations (eg, those with visual impairment and mobility-related limitations) to determine if such an intervention could reduce fall-related events. However, future designs will need to consider comparing historical fall frequency records to future intervention fall frequencies rather than using a control condition if it could pose a fall risk to these populations. Furthermore, we did not include Cohen κ, as a measure of interrater reliability. In addition, the lack of a validated questionnaire, such as Yang et al [27], are methodical limitations and should be included in future studies. In addition, given the emphasis on younger adults in this study, it is unknown whether the striping intervention’s impact is greater in younger versus older adults. Finally, since the steps used here had interstep variations in both riser heights and tread depths, future observational designs could assess these 2 factors independently to determine the impact of each on fall risk.

### Future Directions

Given the considerable negative impact of falls on public health, continued research is necessary to improve safety on stairways. In addition, programs, such as educational campaigns, could be used to raise awareness of factors that contribute to falls [28] and perhaps to help motivate small actions, such as painting stripes on problematic stairways that could have a big impact on public health and provides support for scaling up effective public health interventions for long-term population health benefits.

### Conclusions

This study highlights the importance of addressing interstep variations in stairways to reduce the risk of fall-related events. By understanding the factors that contribute to fall risk and implementing cost-effective interventions, we can work toward creating safer environments for everyone. The findings of this study suggest that contrast enhancement (ie, striping) may be a simple and effective way to reduce the risk of falls associated with interstep variations, highlighting the potential for this approach to make a significant impact on fall prevention efforts.

## Data Availability

The datasets generated during and/or analyzed during this study are not publicly available since the video data are not deidentified.
